# Identification of novel biomarker and therapeutic target candidates for acute intracerebral hemorrhage by quantitative plasma proteomics

**DOI:** 10.1186/s12014-017-9149-x

**Published:** 2017-04-26

**Authors:** Guo-chun Li, Lina Zhang, Ming Yu, Haiyu Jia, Ting Tian, Junqin Wang, Fuqiang Wang, Ling Zhou

**Affiliations:** 1 College of Medicine and Life Sciences, Nanjing University of Chinese Medicine, Nanjing, 210023 People’s Republic of China; 2 The Third Hospital of Zhangzhou, Zhangzhou, 363005 People’s Republic of China; 3 Department of Neurology, Affiliated Hospital of Jiangsu University, Zhenjiang, 212001 People’s Republic of China; 4 School of Public Health, Nanjing Medical University, Nanjing, 211166 People’s Republic of China

**Keywords:** Biomarkers, Acute intracerebral hemorrhage, Proteomics

## Abstract

**Background:**

The systematic mechanisms of acute intracerebral hemorrhage are still unknown and unverified, although many recent researches have indicated the secondary insults. This study was aimed to disclose the pathological mechanism and identify novel biomarker and therapeutic target candidates by plasma proteome.

**Methods:**

Patients with AICH (n = 8) who demographically matched healthy controls (n = 4) were prospectively enrolled, and their plasma samples were obtained. The TMT-LC–MS/MS-based proteomics approach was used to quantify the differential proteome across plasma samples, and the results were analyzed by Ingenuity Pathway Analysis to explore canonical pathways and the relationship involved in the uploaded data.

**Results:**

Compared with healthy controls, there were 31 differentially expressed proteins in the ICH group (*P* < 0.05), of which 21 proteins increased while 10 proteins decreased in abundance. These proteins are involved in 21 canonical pathways. One network with high confidence level was selected by the function network analysis, in which 23 proteins, P38MAPK and NFκB signaling pathways participated. Upstream regulator analysis found two regulators, IL6 and TNF, with an activation *z*-score. Seven biomarker candidates: APCS, FGB, LBP, MGMT, IGFBP2, LYZ, and APOA4 were found. Six candidate proteins were selected to assess the validity of the results by subsequent Western blotting analysis.

**Conclusion:**

Our analysis provided several intriguing pathways involved in ICH, like LXR/RXR activation, acute phase response signaling, and production of NO and ROS in macrophages pathways. The three upstream regulators: IL-6, TNF, LPS, and seven biomarker candidates: APCS, APOA4, FGB, IGFBP2, LBP, LYZ, and MGMT were uncovered. LPS, APOA4, IGFBP2, LBP, LYZ, and MGMT are novel potential biomarkers in ICH development. The identified proteins and pathways provide new perspectives to the potential pathological mechanism and therapeutic targets underlying ICH.

**Electronic supplementary material:**

The online version of this article (doi:10.1186/s12014-017-9149-x) contains supplementary material, which is available to authorized users.

## Background

Intracerebral hemorrhage (ICH) caused by ruptured cerebral blood vessels accounting for 10–20% of all strokes is the most devastating and the least treatable subtype of hemorrhagic stroke [1]. The 30-day mortality of ICH is reported to be 30–55%, nearly half of deaths in the acute stage, especially in the first 48 h, and approximately 80% of survivors are left with severe neurological deficits [2]. Compared with white populations of European origin, Chinese have a higher proportion of ICH. However, the ongoing medical and surgical technological advances have not revealed an effective therapy for ICH. Therefore, it is urgent to explore the mechanisms underlying ICH and to identify the novel therapeutic targets so that we can improve the outcomes of ICH and develop new drugs for it.

Current researches have indicated that brain injury of ICH is primarily related to the secondary effects involving cerebral edema, inflammation autophagy, blood brain-barrier (BBB) disruption, cellular necrosis and apoptosis [3]. However, there has not been any confirmation study of ICH with human so far. Most experiments are undertaken with rodents, not entirely comparable to human ICH [4]. In addition, most people attempt to validate one or a few candidates rather than a panel of potential ICH biomarkers. In recent years, although gene expression profiling of perihematomal tissue after ICH has been performed in animal models as well as in human ICH samples [5], it should be noted that genomic expression and protein levels do not match in some cases [6]. Furthermore, proteins are the most informative biomolecules to advance the field, because not only are they the ultimate gene function performers, but also the direct manifestation of complexity and variability of life as well as the major drug targets of many diseases [6]. The proteins identified in recent researches provide essential information about the target proteins involved in multiple genetic interactions, with a wide range of clinical phenotypes in specific pathological pathways [7, 8]. Quantitative proteomics including isobaric tag for relative and absolute quantitation (iTRAQ) and tandem mass tag (TMT) have been a popular profiling approach in protein biomarker discovery and protein alterations quantification so far [9]. Quite a few researches have successfully obtained significant pathological insights once this technology was applied to brain tissue and cerebrospinal fluid (CSF) [10]. Collection of blood plasma, compared with tissue and CSF, is inexpensive and minimally invasive, and plasma proteins contain a wealth of information about the overall pathophysiological conditions of patients [11]. To date, no comprehensive proteomic analyses of plasma related to ICH has been reported. Therefore, a proteomics profiling of plasma in ICH will complete the pathological mechanisms of ICH and discover the therapeutic target candidates to monitor and improve the prognosis of this disease. In this follow-up study, a TMT-LC–MS/MS-based proteomics approach was used to quantify the differential proteome across plasma samples from ICH patients to explore possible mechanisms and potential therapeutic targets involved in ICH.

## Methods

### Ethics statement

The Ethics Committee of Jiangsu Province Hospital of TCM (Affiliated Hospital of Nanjing University of Chinese Medicine) approved the study protocol. Written informed consent was given by all participates or legal guardians.

### Patient recruitment and sample collection

A total of 12 people were invited to participate in this study. There were 8 ICH patients and 4 demographically matched healthy controls (Table 1). The mean age of ICH patients and controls was 61.6 years (from 56 to 69) and 63.3 years (from 56 to 71) respectively, and 50% were male in both groups. The fasting blood samples were obtained from 8 ICH patients on admission at Affiliated Jiangbin Hospital of Jiangsu University and Affiliated Hospital of Nanjing University of Chinese Medicine (Jiangsu Province Hospital of Traditional Chinese Medicine) in China between 2014 and 2015. The other 4 blood specimens were collected from healthy control subjects at the same time. The following inclusion criteria applied to the patients of the present study: the patients should be consistent with the diagnostic criteria of acute cerebral hemorrhage, admitted within 48 h with stroke onset, and willing and able to timely be referred in accordance with the requirements of the program. Patients were excluded if they were accompanied with transient cerebral ischemia, brain infarction or subarachnoid hemorrhage; their cerebral hemorrhage was caused by blood diseases, tumors or trauma; patients died within 24 h after admission or merged with serious primary diseases. Patients could not cooperate with the inspection because of disabilities included in the legislation framework.

**Table 1:** Experimental protocol and basic characteristics in AICH patients (n = 8) and healthy controls (n = 4) group

Block	Tissue	Disease or normal	Patient number	Age/gender	District and hospital	TMT labeling reagent ID	TMT labeling code
1	1	Normal	1	66/F	Nanjing	1	126
1	2	AICH	2	61/F	Nanjing	1	127
1	4	AICH	3	65/F	Nanjing	1	129
		Reference				1	131
2	6	Normal	4	60/M	Nanjing	2	126
2	7	AICH	5	56/M	Nanjing	2	127
2	9	AICH	6	58/M	Nanjing	2	129
		Reference				2	131
3	11	Normal	7	71/M	Zhenjiang	3	126
3	12	AICH	8	69/M	Zhenjiang	3	127
3	14	AICH	9	66/M	Zhenjiang	3	129
		Reference				3	131
4	16	Normal	10	56/F	Zhenjiang	4	126
4	17	AICH	11	61/F	Zhenjiang	4	127
4	19	AICH	12	57/F	Zhenjiang	4	129
		Reference				4	131

Reference represents a reference sample, i.e., a mixed sample of all plasma samples by internal standard method

*AICH* acute intracerebral hemorrhage, *F* female, *M* male

Whole blood (2 ml) was collected from study subjects. Altogether 12 plasma samples were collected, and K_2_-EDTA was used as the anti-coagulant agent. The blood samples were then centrifuged at 1500 g for 10 min at room temperature. The resulting plasma was aliquotted and stored at −80 °C before use. None of the samples were thawed more than twice before being analyzed.

### Plasma sample preparation and purification

Since disease biomarkers in the blood, which are present at relatively low concentrations, are usually masked by high-abundance proteins, and their signals are weak or even disappear in the mass spectrum [12, 13], Agilent Human 14 Multiple Affinity Removal System (Agilent, USA) was utilized to deplete high-abundant proteins according to the manufacturer’s instructions, including albumin, IgG, antitrypsin, IgA, transferrin, haptoglobin, fibrinogen, ɑ2-macroglobulin, ɑ1-acid glycoprotein, IgM, apolipoprotein AI, apolipoprotein AII, complement C3, and transthyretin. 100 μl of depleted plasma from ICH or normal samples were added into 400 μl of phosphate-buffered saline, 500 μl of ice-cold acetone, 10% trichloroacetic acid (TCA) solution, and then precipitated at −20 °C. The resulting supernatant was removed and the pellets were washed thrice with ice-cold acetone and re-dissolved in 150 μl of urea lysis buffer (8 M urea, 75 mM NaCl, 50 mM Tris, pH 8.2, 1% (V/V) EDTA-free protease inhibitor cocktail) and stored at −80 °C. The protein concentrations were measured by using the Bradford assay. Specific removal of 14 high-abundant proteins depletes approximately 94% of total protein mass from human plasma. The low-abundant proteins in the flow-through fractions can be studied. Removal of high-abundant proteins enables improved resolution and dynamic range for liquid chromatography/mass spectrometry (LC/MS).

### Protein digestion and TMT labeling

One microgram of proteins from normal or ICH samples was reduced for 1 h at 60 °C with 10 mM DTT, and was alkylated with freshly prepared 55 mM iodoacetamide (IAM) at room temperature in darkness for 40 min. Then, the proteins were digested with trypsin overnight at 37 °C by the following buffer: 8 M urea, 75 mM NaCl, 50 mM Tris, pH 8.2, 1% (V/V) EDTA free protease inhibitor cocktail, and the tryptic peptides were desalted and dried in vacuo (Speed Vac, Eppendorf). Subsequently, 100 µg of each sample was labeled with the reagent in accordance with the manufacturer,s protocol (Pierce, Rockford, IL, USA). After 2 h of TMT labeling, 5 µl of 5% hydroxylamine was added to each tube and incubated at room temperature for 15 min. Afterwards, the samples were mixed at the ratio of 1:1 based on total peptide amount, which was obtained by running an aliquot of the labeled sample in a conventional LC–MS/MS. Consequently, peptide expression of each reporter ion was normalized by the average intensity of the corresponding reporter ion (assuming the total intensities for each reporter ion were the same) in the data analysis process.

### Peptide fractionation with SCX chromatography

Labeled peptide mixtures were reconstituted in solution (10 mM NH_4_COOH, 5% ACN, pH 2.7) and fractionated by strong-cation exchange (SCX) columns (1 mm ID × 10 cm packed with Poros 10 S, DIONEX, Sunnyvale, CA, USA), which were installed on the UltiMate^®^ 3000 HPLC system. The following two buffers carried out the separation. Mobile phase A was 5 mM ammonium formate, 5% ACN (pH 2.7); mobile phase B was 800 mM ammonium formate, 5% ACN (pH 2.7). The gradient for SCX was 0–30% B for 21 min, 30–56% B for 7 min, 56–100% B for 1 min, 100% B for 3 min, 100–0% B for 1 min and 0% B for 20 min before the next run. Twenty fractions in total were collected from SCX separation and lyophilization.

### Mass spectrometry analysis

The reversed phase chromatography was performed with a Nano ACQUITY UPLC system on the LTQ-Orbitrap instrument (Thermo Fisher, USA) via a nanospray source. The reversed-phase C18 column (75 μm i.d. × 15 cm, 1.7 μm, 130 Å; BEH) was utilized with buffer A (2% ACN with 0.5% acetic acid) and buffer B (80% ACN with 0.5% acetic acid), respectively. The 193 min linear gradient elution procedure was as follows: 4–9% buffer B for 3 min, 9–33% buffer B for 170 min, 33–50% buffer B for 10 min, 50–100% buffer B for 1 min, 100% buffer B for 8 min, 100–4% buffer B for 1 min. To analyze the proteins of plasma, eight most intense ions for collision-induced dissociation (CID) fragmentation were selected after mass spectrum scanning. CID was performed with 35% normalized collision energy. The most intense ions from MS2 scan were selected and activated by using the higher energy collision-induced dissociation (HCD)-MS3.

### Protein identification and quantification

The raw files obtained from precursor ions were analyzed with MaxQuant (version 1.2.2.5). Sequences search parameters were as follows: precursor mass tolerance was 20 parts per million (ppm); product ion mass tolerance was 0.5 Da; enzyme was trypsin with two maximum missed cleavages; cysteine carbamidomethylation (+57.02146 Da), N-terminus and lysine modification with TMT reagent adducts (+229.162932 Da) were set as a fixed modifications; methionine oxidation (+15.99492 Da) was set as a dynamic modification. The false discovery rates (FDR) of peptide and protein identification, which were estimated by searching against the database, were set <1%. Only peptides with at least six amino acids in length were considered to be successfully identified. The ratios of relative protein abundance between two groups were calculated by intensities of TMT reagent reporter ion from HCD spectra.

### Western blot analyses

A standard western blotting analysis was used to investigate the protein expression levels after protein components were collected. Protein lysates from the plasmas of healthy controls (n = 8) and ICH patients (n = 8) were boiled in SDS-sample buffer for 5 min and then subjected to 8% SDS-polyacrylamidegel electrophoresis and transferred to PVDF (polyvinylidenefluoride) membranes (Bio-Rad). The membranes were then blocked for 2 h in 5% milk-Tris-Buffered Saline Tween-20 (TBST) at room temperature, and incubated overnight at 4 °C either with the monoclonal antibodies (Cell Signaling Technology, USA) of anti-APOA4, anti-IGFBP2, anti-LBP, anti-MGMT, anti-FGB or anti-APCS. The membranes were washed four times in TBST and incubated with the appropriate horseradish peroxidase-conjugated secondary antibodies at room temperature for 2 h. Blots were visualized by enhanced chemiluminescence (Thermo, USA) and analyzed by a scanning densitometer with the molecular analysis software FluorChem M system (Protein Simple, USA) [14].

### Bioinformatics analysis

#### Statistical analysis

SPSS for Windows, Version 20.0 (Armonk, NY: IBM Corp.) was used for all statistical analyses. Univariate ANOVA (two-way analysis of variance with block design or one-way analysis of variance) was applied to compare the protein abundance among different groups. Protein abundance of control group was used for a reference to calculate the fold-change; changes of 1.3 or higher and the *P* value <0.05 were considered significant.

#### Ingenuity Pathway Analysis

In order to further understand the biological significance of differentially expressed plasma proteins, Ingenuity Pathway Analysis (IPA; Ingenuity^®^ Systems, www.Ingenuity.com/) was used to analyze canonical pathways and relationship of the uploaded data. Right-tailed Fisher’s exact test was used to calculate a *P* value to determine the significance of each canonical pathway, and *P* value <0.05 was meaningful. Disease and functional protein networks and upstream regulator analysis with differently expressed proteins were presented, along with a Z-score. The Z-score ≥2 or ≤−2 was considered significant activation or significant inhibition respectively.

## Results

### Quantification of plasma proteins in healthy control and ICH samples

**Fig. 1:**
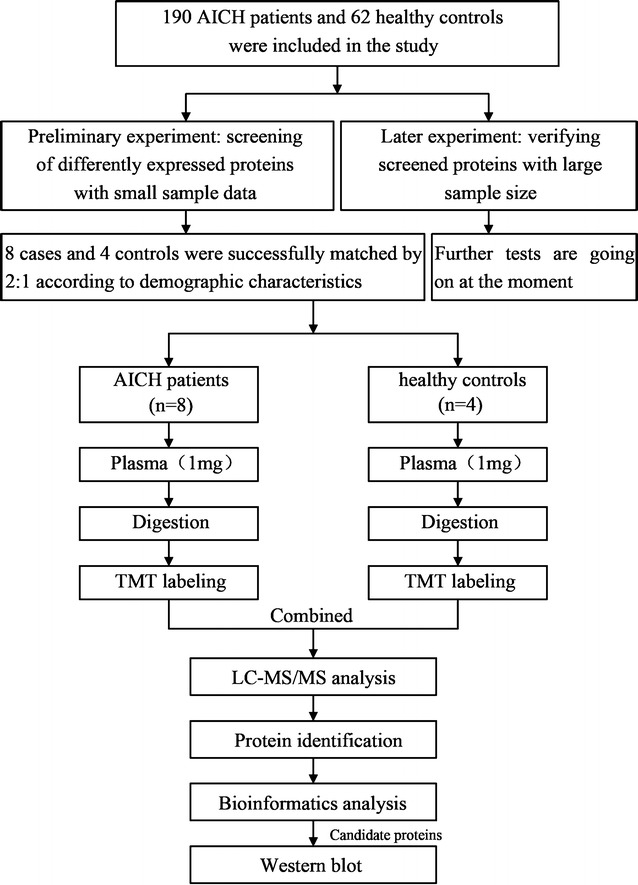
The experimental profile

Tandem mass spectrometry and TMT-labeled relative quantification proteomics were employed to screen differentially expressed proteins between ICH patients and healthy controls. The experimental workflow of this study is shown in Fig. 1. A total of 414 proteins and 3886 peptides were identified. Based on reporter ions, 411 proteins and 3165 peptides were quantified. The overall calibration factors and intensity distributions for all reporter ions were shown in Additional file 1: Figure S1. Compared with health control (NC) group, there were 31 differentially expressed proteins in ICH group (fold change > 1.2 or fold change < −1.2, *P* < 0.05), of which 21 proteins increased remarkably and the other 10 decreased in abundance (Table 2). The average fold-change for differentially expressed proteins between AICH and NC group was calculated to make a comparison.

**Table 2:** List of proteins with different abundance in AICH patients ($\overline{\text{x}} \,{ + }\,{\text{sd}}$)

No.	UniProt accession	Symbol	Entrez gene name	Abundance in AICH	Abundance in NC	Fold change	95% CI for fold change	*P* value
1	A0A024R035	C9	Complement component 9	1.087 ± 0.109	0.785 ± 0.204	1.256 ± 0.258	1.256 (1.092, 1.420)	0.016
2	A0A024R4F1	ENO1	Enolase 1, (ɑ)	1.386 ± 0.582	0.952 ± 0.521	1.304 ± 0.608	1.304 (0.918, 1.690)	0.038
3	A0A087X271	CNN2	Calponin 2	1.023 ± 0.165	1.404 ± 0.134	−1.268 ± 0.278	−1.268 (−1.560, −0.977)	0.008
4	A0A0C4DFQ9	DCUN1D2	DCN1, defective in cullin neddylation 1, domain containing 2	0.571 ± 0.394	2.086 ± 0.497	−4.096 ± 4.251	−4.096 (−8.557, 0.367)	0.013
5	A2VCK8	TMSB10/TMSB4X	Thymosin beta 10	1.418 ± 0.601	0.634 ± 0.262	1.824 ± 0.994	1.824 (1.193, 2.456)	0.049
6	B4E1N5	APOL1	Apolipoprotein L, 1	1.197 ± 0.287	0.895 ± 0.266	1.225 ± 0.342	1.225 (1.007, 1.442)	0.028
7	D6RB59	EXOC3	Exocyst complex component 3	1.280 ± 0.023	0.433 ± –	2.304 ± 1.130	2.304 (−0.502, 5.109)	0.022
8	E9PN51	NDUFS8	NADH dehydrogenase (ubiquinone) Fe-S protein 8, 23 kDa (NADH-coenzyme Q reductase)	0.906 ± 0.009	0.364 ± –	1.995 ± 0.862	1.995 (−0.146, 4.135)	0.013
9	F2RM37	F9	Coagulation factor IX	1.005 ± 0.100	0.750 ± 0.236	1.227 ± 0.258	1.227 (1.064, 1.391)	0.038
10	F8VV32	LYZ	Lysozyme	1.031 ± 0.265	1.425 ± 0.364	−1.321 ± 0.365	−1.321 (−1.553, −1.089)	0.017
11	G3V2H1	MPP5	Membrane protein, palmitoylated 5 (MAGUK p55 subfamily member 5)	0.586 ± 0.174	1.937 ± 0.301	−2.699 ± 1.559	−2.699 (−4.335, −1.063)	0.007
12	H7C267	PES1	Pescadillo ribosomal biogenesis factor 1	0.497 ± 0.023	2.735 ± –	−4.005 ± 2.608	−4.005 (−10.484, 2.475)	0.008
13	H7C4S2	HBP1	HMG-box transcription factor 1	0.999 ± 0.020	0.398 ± –	2.006 ± 0.872	2.006 (−0.159, 4.171)	0.025
14	L8EB32	COL14A1	Collagen, type XIV, ɑ 1	1.054 ± 0.411	2.574 ± 0.454	−2.162 ± 1.206	−2.162 (−3.428, −0.896)	0.005
15	P00739	HPR	Haptoglobin-related protein	1.106 ± 0.505	0.440 ± 0.096	2.007 ± 1.185	2.007 (1.254, 2.760)	0.046
16	P02671	FGA	Fibrinogen ɑ chain	1.050 ± 0.230	0.694 ± 0.114	1.342 ± 0.375	1.342 (1.103, 1.580)	0.026
17	P06396	GSN	Gelsolin	0.947 ± 0.096	1.367 ± 0.326	−1.321 ± 0.274	−1.321 (-1.494, −1.147)	0.003
18	P06727	APOA4	Apolipoprotein A-IV	0.796 ± 0.115	1.418 ± 0.334	−1.562 ± 0.467	−1.562 (−1.859, −1.266)	<0.001
19	P18065	IGFBP2	Insulin-like growth factor binding protein 2, 36 kDa	1.230 ± 0.238	1.742 ± 0.612	−1.327 ± 0.343	−1.327 (−1.687, −0.966)	0.041
20	P18428	LBP	Lipopolysaccharide binding protein	1.180 ± 0.309	0.700 ± 0.167	1.457 ± 0.504	1.457 (1.137, 1.778)	0.005
21	P19652	ORM2	Orosomucoid 2	0.858 ± 0.176	0.625 ± 0.110	1.248 ± 0.304	1.248 (1.055, 1.442)	0.036
22	Q08830	FGL1	Fibrinogen-like 1	0.992 ± 0.162	0.543 ± 0.087	1.552 ± 0.491	1.552 (1.036, 2.067)	0.047
23	Q53S54	CUL3	Cullin 3	1.333 ± 0.465	0.497 ± 0.145	2.123 ± 1.130	2.123 (1.254, 2.992)	0.008
24	Q6LDD1	MGMT	*O*-6-methylguanine-DNA methyltransferase	1.081 ± 0.005	0.315 ± –	2.618 ± 1.402	2.618 (−0.863, 6.100)	0.005
25	Q6ZW64	IGH	Immunoglobulin heavy locus	1.175 ± 0.453	0.528 ± 0.239	1.816 ± 0.942	1.816 (1.218, 2.415)	0.009
26	V9HVY1	FGB	Fibrinogen beta chain	1.010 ± 0.153	0.719 ± 0.145	1.270 ± 0.282	1.270 (1.091, 1.449)	0.015
27	V9HW21	CA2	Carbonic anhydrase II	1.296 ± 0.205	0.901 ± 0.071	1.292 ± 0.286	1.292 (1.072, 1.512)	0.007
28	V9HWF6	ORM1	Orosomucoid 1	0.911 ± 0.235	0.472 ± 0.121	1.622 ± 0.622	1.622 (1.226, 2.017)	0.002
29	V9HWI6	GC	Group-specific component (vitamin D binding protein)	0.962 ± 0.223	1.553 ± 0.497	−1.488 ± 0.456	−1.488 (−1.778, −1.198)	0.008
30	V9HWP0	APCS	Amyloid P component, serum	1.067 ± 0.170	0.811 ± 0.107	1.211 ± 0.238	1.211 (1.059, 1.362)	0.048
31	X6RJP6	TAGLN2	Transgelin 2	1.074 ± 0.289	0.825 ± 0.176	1.202 ± 0.335	1.202 (0.988, 1.415)	0.017

Positive value indicates up-regulation and negative value indicates down-regulation in fold change column; AICH, intracerebral hemorrhage; CI, confidence interval for mean; – indicates data missing because of no enough sample size

### Gene ontology (GO) analysis

The Gene Ontology (GO) data were used to analyze the subcellular location and the function of the differentially expressed proteins. Figure 1 displays that 16 proteins (approximately 51.61%) have been specified as extracellular space proteins. About 19.35% of the remaining proteins with subcellular location annotation were cytoplasm proteins, and 12.9% proteins were located in nucleus. The largest proportion of the differentially expressed proteins was represented by binding (41.9%), followed by proteins with catalytic activity (32.3%), transporter activity (12.9%), and reporter activity (12.9%). Regarding the biological process categories, the majority of proteins were involved in metabolic process (45.2%), cellular process (38.7%), response to stimulus (29.0%), localization (29.0%) and multicellular organismal process (19.4%). Hence, extracellular space, binding, and cellular process were the major subcellular location, molecular function and cellular process of the proteins. And the overall functional distributions of the non-significantly expressed genes were similar to DE proteins (Additional file 2: Figure S2).

### IPA analysis of the proteins detected in ICH group

In order to understand the biological significance of the 31 differentially expressed proteins in ICH better, the proteins were subjected to IPA software for further analysis of significantly involved molecular process. According to the Ingenuity Pathways Knowledge Base, IPA provided important information, such as canonical pathways, protein interaction networks, and upstream regulators. The canonical pathways along with up- and down-regulated proteins in each pathway were displayed in the bar chart of differentially expressed proteins. IPA analysis revealed that 10 proteins (APOA4, APOL1, C9, FGA, GC, HPR, LBP, LYZ, ORM1, ORM2) were in the positive Liver X receptor and retinoic acid X (LXR/RXR) activation pathway; 7 proteins (APCS, C9, FGA, FGB, LBP, ORM1, ORM2) in the positive acute phase response signaling pathway; 5 proteins (APOA4, APOL1, LYZ, ORM1, ORM2) in the positive production of nitric oxide (NO) and reactive oxygen species in macrophages (ROS) pathway.

**Fig. 2:**
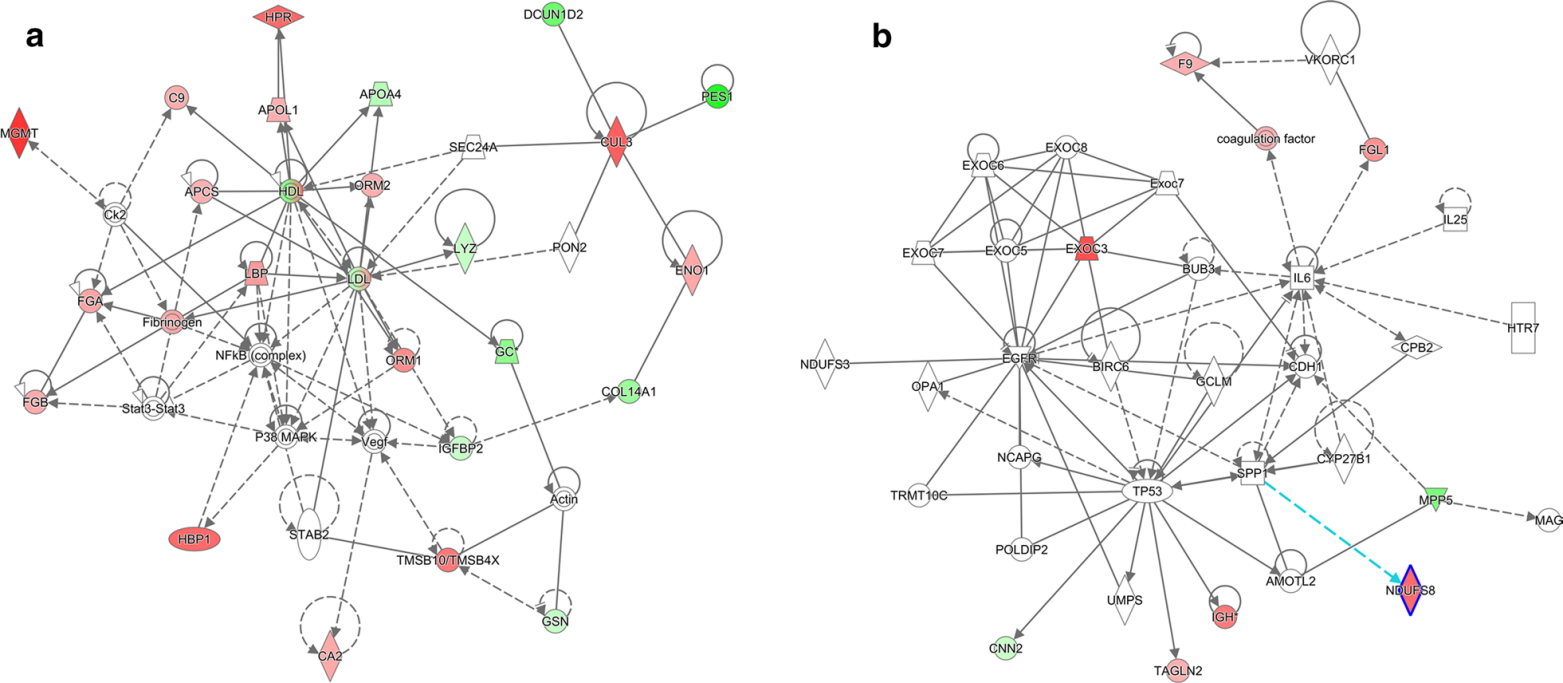
The top 2 diseases and disorders networks identified by IPA analysis. **a** Metabolic disease, cell-to-cell signaling and interaction, renal damage network. **b** Cellular growth and proliferation, cancer, organismal injury and abnormalities network. *Red* and *green* indicate up- and down-regulation, respectively, of the proteins. Intensity of the color reflects the extent of differential expression. *Dot lines* represented indirect interaction; *solid lines* represented direct interaction; and *arrows* represented the direction of interaction

 These proteins were also associated with functional network of metabolic diseases, cell-to-cell signaling and interaction. There were 23 proteins in the network (APCS, APLA4, APOL1, C9, CA2, COL14A1, CUL3, DCUN1D2, ENO1, FGA, FGB, GC, GSN, HBP1, HPR, IGFBP2, LBP, LYZ, MGMT, ORM1, ORM2, RES1, TMSB10/TMSB4X), and they were regulated by p38 Mitogen activated protein kinase (P38MAPK) as well as nuclear transcription factor κB (NFκB) (Fig. 2). The molecules in network and the score of network clustering were reported in Table 3.

**Table 3:** Major networks and associated proteins obtained by IPA analysis of differentially expressed ICH proteins

Top diseases and functions	Molecules in network	Score	Focus molecules
Metabolic disease, cell-to-cell signaling and interaction, renal damage	Actin, *APCS↑*, *APOA4↓*, *APOL1↑*, *C9↑*, *CA2↑*, Ck2, *COL14A1↓*, *CUL3↑*, *DCUN1D2↓*, *ENO1↑*, *FGA↑*, *FGB↑*, Fibrinogen, *GC↓*, *GSN↓*, *HBP1↑*, HDL, *HPR↑*, *IGFBP2↓*, *LBP↑*, LDL, *LYZ↓*, *MGMT↑*, NFB (complex), *ORM1↑*, *ORM2↑*, P38MAPK, *PES1↓*, PON2, SEC24A, STAB 2, Stat3-Stat3, *TMSB10/TMSB4X↑*, Vegf	64	23

The italicised proteins are identified in our data, and their expression is shown by arrows: up-regulation (↑) and down-regulation (↓)

**Fig. 3:**
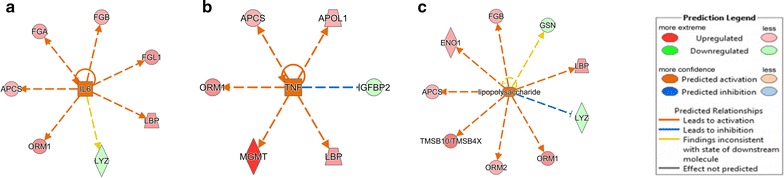
Upstream regulator analysis of differentially expressed proteins in ICH. IL-6, TNF and LPS were predicted to be activated as determined by IPA. **a**, **b**, **c** represent 3 upstream regulators (IL-6, TNF and LPS) and their target molecules for prediction in our dataset respectively

Another meaningful research that we performed with IPA was upstream regulator analysis, which could predict the activation or inhibition of upstream regulators based on the differently expressed proteins identified in the present study so that the biological activities occurring in the ICH plasmas could be better understood. In our data, 99 upstream regulators of proteins have been discovered with a *P* value <0.05, three of which were of activation *z*-score, including IL6 (*z*-score 2.026), TNF (*z*-score 2.371), and lipopolysaccharide (LPS) (*z*-score 2.219). They were considered to be target candidates for drug treatment. Theoretically, any chemical capable of inhibiting the expression of the three cytokines had the potential to treat ICH by preventing its progress and deterioration. The network analyses of the three upstream regulators and their target molecules for prediction in our dataset were shown in Fig. 3.

To identify the potential biomarkers, we used the IPA biomarker filter to match the uploaded proteins with the list of biomarkers known for disease profiles. Some parameters settings in the IPA, with the related available options, were as follows: (1) Human species; (2) All molecule types; (3) Blood; (4) All biomarkers and disease applications (including cardiovascular disease, hematological disease, immunological disease, inflammatory disease, metabolic disease, and neurological disease). Seven proteins (APCS, APOA4, FGB, IGFBP2, LBP, LYZ, MGMT) were finally obtained as predicted markers for ICH shown in Table 4.

**Table 4:** Selected candidate biomarkers by IPA biomarker filter analysis

UniProt accession	Symbol	Entrez gene name	Location	Exp fold change
V9HWP0	APCS	Amyloid P component, serum	Extracellular space	1.211
P06727	APOA4	Apolipoprotein A-IV	Extracellular space	−1.562
V9HVY1	FGB	Fibrinogen beta chain	Extracellular space	1.270
P18065	IGFBP2	Insulin-like growth factor binding protein 2, 36 kDa	Extracellular space	−1.327
P18428	LBP	Lipopolysaccharide binding protein	Plasma membrane	1.457
F8VV32	LYZ	Lysozyme	Extracellular space	−1.321
Q6LDD1	MGMT	*O*-6-methylguanine-DNA methyltransferase	Nucleus	2.618

### Validation of proteomics results by Western blotting

**Fig. 4:**
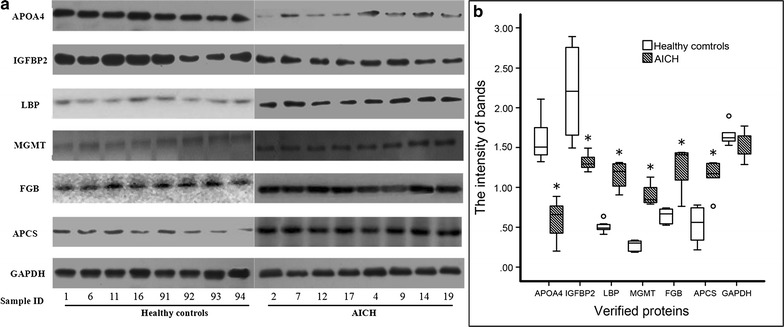
Western blotting analysis of APOA4, IGFBP2, LBP, MGMT, FGB and APCS from healthy controls and AICH. GAPDH is the reference group by internal standard method. **a** Western blotting analysis of the six proteins. **b** The intensity of each band was measured using imaging analysis. *P < 0.05 versus control

Western blotting analysis using GAPDH as an internal reference was performed for six important proteins (APOA4, IGFBP2, LBP, MGMT, FGB and APCS) to validate the iTRAQ results of the identified proteins. Consistent with iTRAQ proteomics results, the intensity of bands by Western blotting of six proteins significantly changed in the plasma of AICH compared with healthy control subjects (Fig. 4, *P* < 0.05).

## Discussion

In this study, proteins extracted from plasmas of 8 ICH patients and 4 NC patients have been quantified by tandem-mass-TMT tagging labeling technology. Different expressions of 31 proteins in all were discovered, of which 21 were up-regulated, accounting for around 1/3. As far as we know, this is the first comprehensive study to analyze ICH plasmas from each patient individually, in which high-throughput protein identification and quantification by MS were used to identify ICH biomarker.

Primary damage of ICH occurs within a few minutes to several hours since the onset of bleeding. It is mainly mechanical damage caused by hematoma with mass effect. However, secondary damage of ICH, in most cases, is due to many parallel pathological pathways including: (1) cytotoxicity of blood, (2) hypermetabolism, (3) excitotoxicity, (4) spreading depression, (5) oxidative stress and inflammation. The canonical pathway analysis shows that 12 out of 31 proteins activate LXR/RXR pathway and participate in the acute phase response. They are the production of NO and ROS by macrophages, too. LXR/RXR is involved in the regulation of lipid metabolism, inflammation, cholesterol and bile acid catabolism [15]. Acute phase response is a rapid inflammatory response triggered by tissue injury, infection, or immunological disorders [16]. In the process of inflammation, the production of NO pro-inflammatory mediators in macrophages is attributed to the inducible form of nitric oxide synthase (iNOS). In addition, the microbicidal properties of macrophages are dependent on the production of reactive oxygen species (ROS). High level of NO and ROS causes oxidative stress, inflammatory reaction, eventually leading to tissue damage [17]. It has been reported that NO in ICH would result in secondary neuronal damage and aggravate tissue injury in the central nervous system, while ROS may be a major trigger of NLPR3 inflammasome activation following ICH [18, 19]. The activation of three canonical pathways supports the momentous effect of inflammation in ICH as previous researches reported. Accordingly, we assume that the inflammatory response in ICH may be reduced effectively by inhibiting activation of the above signaling pathways.

IPA created an interaction network among the differentially expressed proteins, meanwhile, other signaling proteins revealed the interactions between disease and function. In the present study, these proteins are associated with metabolic diseases, cell-to-cell signaling and renal damage in which p38MAPK and NFκB are involved. Amazingly, no ICH related networks have been confirmed based on the existing IPA knowledge database, illustrating that the identified proteins have not been previously reported in a network with functions related to ICH. For all that, p38MAPK and NFκB are recommended as key regulators in the network, for it regulated the majority (23/31) of the proteins in this research. P38MAPKs, as one MAPK family member, is responsible for signal transmission between extracellular space to the nucleus, leading to biology reaction of cells [20]. A number of evidences demonstrate that p38MAPK signaling and NFκB are important inflammatory pathways in ICH through various mechanisms. Zhao et al. [21] found that p38MAPK and NFκB were activated in 24 h in animal model of ICH, and the use of inhibitor could recede the inflammatory injury after ICH. Meanwhile, the activation of NFκB in microglia/macrophages after ICH would result in the increase of pro-inflammatory cytokines such as TNF-α and IL-1β that might cause brain injury [22]. Together, p38MAPK and NFκB could represent potential therapeutic targets to prevent neurological deficits after ICH.

 IPA analysis found three upstream regulators activated based on the differently expressed proteins. IL-6 is the key upstream regulator of 7 proteins, including FGA, FGB, FGL1, LBP, APCS, ORM1 and LYZ, because 6 out of the 7 proteins were activated by IL-6. TNF activated 5 proteins, including APCS, APOL1, ORM1, MGMT and LBP. LPS is an upstream regulator of 9 proteins, including FGB, ENO1, APCS, TMSB10/TMSB4X, ORM2, ORM1, LYZ and LBP, and IPA showed that 7 out of 9 proteins were activated by LPS. IL-6 and TNF are cytokines that control differentiation, activation, apoptosis and other functions in many cell types [23]. As pro-inflammation cytokines, IL-6 and TNF play important roles in ICH-induced inflammatory injury [24]. Immune system that is activated after ICH induces the release of numerous inflammatory cytokines, such as TNF-α and IL-6, and then the inflammatory responses damage brain tissue and kill plenty of brain cells [25]. Lei et al. [26] used the antibody of TNF to antagonize the inflammation response, which displayed that the antibody gave rise to palliating neuro-inflammation and enhancing functional outcomes in a murine model of ICH. This study confirmed that IL-6 and TNF might be ideal drug targets for ICH. LPS is a form of endotoxin and has a significant effect on many diseases, such as septic shock, sepsis and Th2 inflammatory diseases [27], but its role in ICH has not been completely elucidated so far. LPS combined with CD14 would stimulate the release of inflammatory mediators from monocytes and endothelial cells, and finally cause systemic inflammatory response syndrome [28]. In this study, the upstream regulator of LPS activated 7 proteins shown with arrows in orange (Fig. 3c). The findings suggest that the discovery above is consistent with previous literatures, and LPS is indeed a good drug target candidate for ICH. But, it requires further experiments to test the effect of LPS on ICH and to validate the discovery of this study.

Identification of the putative biomarkers predicts 7 proteins as ICH markers by IPA. The level of 4 proteins (APCS, FGB, LBP, MGMT) increases, while three proteins (APOA4, IGFBP2, LYZ) decrease in ICH plasma samples. APCS is a glycoprotein, belonging to the pentraxin family. As a chaperone, it works on the binding of the encode protein to proteins in the pathological amyloid, and it increases amyloidosis of mice [29]. According to the report, the third etiological subcategory of ICH is the deposition of amyloid proteins in cerebral arteries, causing more blood vessels exudate [30]. Overexpression of APCS in ICH suggests that it may be an etiologic blood biomarker of ICH. FGB is the member of fibrinogen, cleaved by thrombin to form fibrin following vascular injury. Moreover, various cleavage products of fibrinogen and fibrin regulate cell adhesion, spreading and chemotactic activities, and they are mitogens of several cell types [31]. Leira et al. [32] have validated that the fibrinogen is an independent predictor of early neurologic deterioration in ICH patients. LBP is one of the acute phase immunologic response proteins for bacterial infection known to be involved in regulating LPS-dependent monocyte response [33]. As described above, the combination of LPS and CD14 can initiate inflammation response which is mediated by LBP, and many studies indicate that LBP is a marker of acute inflammation in patients with trauma [34]. In this study, the up-regulation of LBP suggests that it may be implicated in the acute inflammation response within 48 h after ICH, thus it could be used as a candidate marker for ICH patients. MGMT is a DNA repair protein that plays a role in cellular defense against mutagenesis and toxicity from alkylating agents [35]. MGMT has been associated with several cancers like colorectal cancer and lung cancer [36], but its function in ICH has not been reported previously, so clinical experiments are needed to verify the results of this research.

Ideal biomarkers should be selectively and highly expressed in ICH patients [37]. However, APOA4, IGFBP2, and LYZ were down-regulated in ICH. APOA4 is a powerful activator of cholesterol transporter in vitro and is involved in antioxidant activity [36]. IGFBP2 is one of the six similar proteins that bind IGF-I and IGF-II with high affinity. Its high expression level promotes the growth of several types of tumors [38]. LYZ is one of the antimicrobial agents found in human milk, kidney and tears. It is of antibacterial activity against a number of bacterial species. But the role of these proteins play in ICH has not been clear, thus further research is needed to elucidate the effect of the three proteins on ICH.

Limitation in this research is the small sample size, although matching method that can reduce sample size is used to control confounding. We will expand the sample size in the following research. Small sample studies may be false negative, but the positive results of the protein are meaningful by verification.

## Conclusion

Advances in neuroimaging and animal models have improved our understanding of the pathophysiology of ICH on molecular level. Most current theories imply a two-phase model of nerve damage: early mechanical damage phase and sub-acute phase characterized by inflammation and edema as response to hemorrhage. Proteomics analysis helped to find 31 plasma proteins which were differently expressed in acute ICH patients. Our analysis provided several novel pathways involved in ICH, like LXR/RXR activation, acute phase response signaling, and production of NO and ROS in macrophages pathways. In the network analysis, p38MAPK and NFκB were known to implicate in progression and deterioration in the secondary damage phase of ICH. In addition, we also discovered 3 upstream regulators including IL-6, TNF and LPS, as well as 7 biomarker candidates including APCS, APOA4, FGB, IGFBP2, LBP, LYZ and MGMT. Among them, IL-6, TNF, APCS, FGB have been identified to promote ICH, but the remaining proteins are novel potential biomarkers which deserved further validation. Therefore, our data provided an unparalleled wealth of knowledge of ICH pathophysiology. The identified proteins and pathways provide new perspectives to the potential pathological mechanism and therapeutic targets underlying ICH.

## Additional files

**Additional file 1: Figure S1.** Calibration factors and overall intensity distributions for TMT labeling.

**Additional file 2: Figure S2.** The GO analysis of 31 differently expressed proteins and all other proteins quantified (those without differential abundance).

## Abbreviations

AICH: acute intracerebral hemorrhage
ICH: intracerebral hemorrhage
TMT: tandem mass tag
CSF: cerebrospinal fluid
TMT-LC–MS/MS: two-dimensional-liquid chromatography tandem mass spectrometry (2D-LC-MS/MS) with tandem mass tagging (TMT)
SCX: strong-cation exchange
CID: collision-induced dissociation
HCD: higher energy collision-induced dissociation
FDR: false discovery rates
IPA: Ingenuity Pathway Analysis
NC: normal controls
GO: gene ontology
NO: nitric oxide
ROS: reactive oxygen species in macrophages
p38MAPK: p38 mitogen activated protein kinase
NFκB: nuclear transcription factor κB
