# Supplementary figures and images for: Identification of novel biomarker and therapeutic target candidates for acute intracerebral hemorrhage by quantitative plasma proteomics
Source: Clin Proteomics. 2017 Apr 26;14:14. doi: 10.1186/s12014-017-9149-x (PMC5406897; doi:10.1186/s12014-017-9149-x)

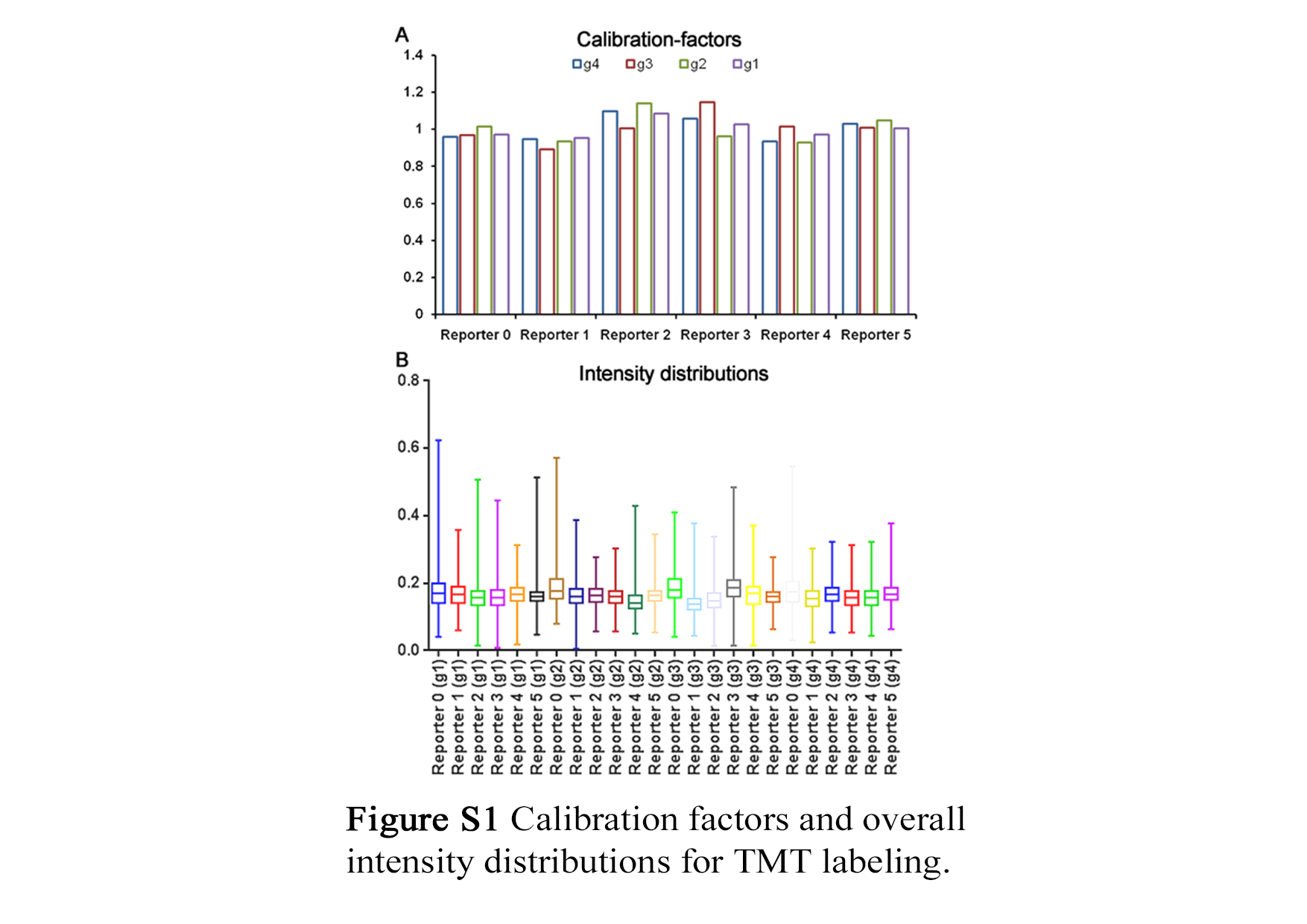

Supplement: Supplementary file 1 — Additional file 1: Figure S1. Calibration factors and overall intensity distributions for TMT labeling. [file 12014_2017_9149_MOESM1_ESM.tif]

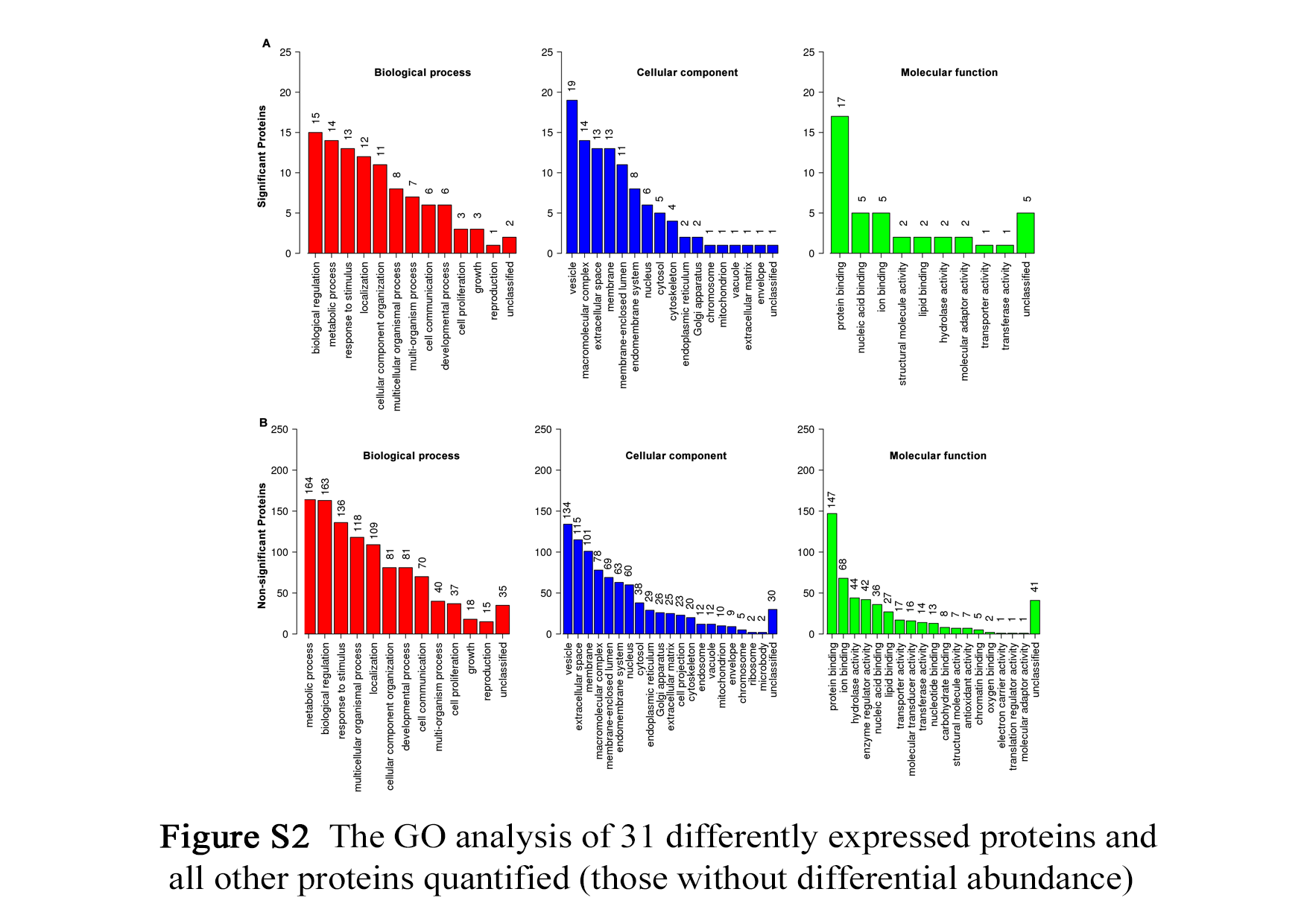

Supplement: Supplementary file 2 — Additional file 2: Figure S2. The GO analysis of 31 differently expressed proteins and all other proteins quantified (those without differential abundance). [file 12014_2017_9149_MOESM2_ESM.tif]
